# Multiple Infections by the Anther Smut Pathogen Are Frequent and Involve Related Strains

**DOI:** 10.1371/journal.ppat.0030176

**Published:** 2007-11-16

**Authors:** Manuela López-Villavicencio, Odile Jonot, Amélie Coantic, Michael E Hood, Jérôme Enjalbert, Tatiana Giraud

**Affiliations:** 1 Univ Paris-Sud, Laboratoire Ecologie, Systématique et Evolution, Orsay, France; 2 CNRS, UMR 8079, Orsay, France; 3 Department of Biology, Amherst College, Amherst, Massachusetts, United States of America; 4 Laboratoire de Pathologie Végétale, INRA BIOGER, Thiverval Grignon, France; University College Cork, Ireland

## Abstract

Population models of host–parasite interactions predict that when different parasite genotypes compete within a host for limited resources, those that exploit the host faster will be selected, leading to an increase in parasite virulence. When parasites sharing a host are related, however, kin selection should lead to more cooperative host exploitation that may involve slower rates of parasite reproduction. Despite their potential importance, studies that assess the prevalence of multiple genotype infections in natural populations remain rare, and studies quantifying the relatedness of parasites occurring together as natural multiple infections are particularly scarce. We investigated multiple infections in natural populations of the systemic fungal plant parasite Microbotryum violaceum, the anther smut of Caryophyllaceae, on its host, Silene latifolia. We found that multiple infections can be extremely frequent, with different fungal genotypes found in different stems of single plants. Multiple infections involved parasite genotypes more closely related than would be expected based upon their genetic diversity or due to spatial substructuring within the parasite populations. Together with previous sequential inoculation experiments, our results suggest that M. violaceum actively excludes divergent competitors while tolerating closely related genotypes. Such an exclusion mechanism might explain why multiple infections were less frequent in populations with the highest genetic diversity, which is at odds with intuitive expectations. Thus, these results demonstrate that genetic diversity can influence the prevalence of multiple infections in nature, which will have important consequences for their optimal levels of virulence. Measuring the occurrence of multiple infections and the relatedness among parasites within hosts in natural populations may be important for understanding the evolutionary dynamics of disease, the consequences of vaccine use, and forces driving the population genetic structure of parasites.

## Author Summary

Infections of one host individual by multiple genotypes of a parasite occur in many natural systems and have major consequences on the evolution of disease severity. Under such multiple infections, the parasite genotypes compete for the host's limited resources, and the faster exploiters will be advantaged over more prudent genotypes, selecting for parasites that cause greater damage and mortality, i.e., having higher virulence. However, when different parasite genotypes within a host are related, a reduction of competitive conflicts between them should lead to more cooperative host exploitation, and thus to lower severity of disease. The occurrence of multiple infections and the relatedness among parasite genotypes within hosts therefore are important to our understanding of diseases, but studies that assess these parameters in nature remain scarce. We investigated multiple infections by the fungus Microbotryum violaceum, responsible for the anther smut disease on the plant Silene latifolia. Multiple infections in natural populations were extremely frequent, with many different genotypes within single host plants. The fungal genotypes found in the different stems of single plants were, however, more related than expected by chance. Together with previous artificial inoculation experiments, these results suggest that M. violaceum actively excludes dissimilar genotypes while tolerating closely related competitors.

## Introduction

It is generally considered that a parasite's optimal level of virulence, i.e., the decrease in host fitness induced by disease that maximizes parasite transmission, depends on several factors, such as mode of transmission, dormancy ability, host availability, and the frequency of occurrence within single hosts of multiple infections by different parasite genotypes [1,2]. The occurrence of multiple infections is theoretically predicted to be an important determinant of virulence evolution because when different parasites compete for limited resources within a single host, more rapid exploitation strategies should win over prudent strategies, thus selecting for parasites that cause greater damage and mortality [2]. However, when related parasites share a host, kin selection should reduce between-genotype competition, resulting in shared host exploitation that is more cooperative [3]. This predicted consequence of kin selection among parasites has been supported by some empirical data [4–7]. The relatedness among parasites can also affect the prevalence of within-host competition if there is competitive exclusion that is dependent upon the genetic distance among parasites [8]. Our understanding of parasite virulence evolution would therefore benefit from measures of the frequency of multiple infections and the relatedness among parasite genotypes within hosts in natural populations. Multiple infections have been reported for several plant parasites [9], including a few diseases causing systemic infections [10,11], for which the competition for resources should be stronger because they invade their whole host. Multiple infections have also been reported for a few animal and human parasites [7,12–14]. In contrast, the relatedness between naturally infecting parasites within plants and animals in natural populations remains largely unexplored (but see [12,14] for human parasites).

In this study, we surveyed several natural populations of the Caryophyllaceae Silene latifolia infected by the anther smut fungus *Microbotryum violaceum.* The *M. violaceum–S. latifolia* system is an important biological model for the study of host–parasite evolution [8,15–18]. The fungus sterilizes the hosts by destroying ovaries and replacing pollen with fungal spores that are then dispersed by natural pollinators of the plant (Figure 1). Multiple infections have been observed in S. latifolia after experimental inoculation by several genotypes of M. violaceum [18,19] and in experimental populations [20], with different fungal genotypes segregating in different stems of the plant. Even more interestingly, experimental inoculations showed that sequential inoculations with more related genotypes led to a higher frequency of multiple infections than sequential inoculation with less related genotypes even though the different strains inoculated alone yielded similar levels of infection [8]. These experimental results strongly suggested that a mechanism of active exclusion exists whereby resident genotypes exclude challenge by distantly related parasites. However, studies in natural populations are required to assess whether this experimental phenomenon was relevant to natural infections.

**Figure 1:**
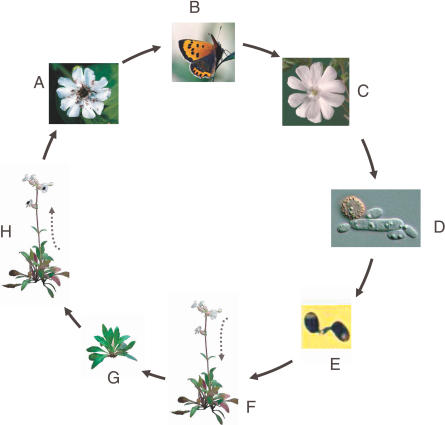
Life Cycle of the Phytopathogenic Fungus M. violaceum Diploid teliospores are produced in the anthers (A) and are transmitted by pollinators (B) onto a healthy plant (C). The teliospores germinate, undergo meiosis, and produce yeast-like sporidia (D). Conjugation takes place on the plant between sporidia of opposite mating types (E). Dikaryotic hyphae grow in the plant (F) and overwinter in vegetative tissues (G). The following year, infection is systemic (H) and all flowers produce teliospores (A).

## Results/Discussion

### Frequent Multiple Infections

We collected one flower bud per branch of every diseased plant in 12 roadside populations of S. latifolia in order to determine the frequency of multiple infections and to assess the degree of parasite relatedness within plants with multiple infections. For each bud, the parasite genotyped from one sporulating anther was tested by using six microsatellite markers and amplified fragment length polymorphism (AFLP).

Multiple infections were extremely frequent: 70% of the 190 diseased plants analyzed carried distinct parasite genotypes in different stems. On average, we detected 2.33 ± 0.5 genotypes per plant, and some plants yielded up to nine different parasite genotypes (Figure 2). In contrast, only a single genotype was detected per flower bud. In most buds, only a single allele was detected at each locus, in accordance with the high degree of homozygosity known to occur in M. violaceum [21], and we never detected more than two alleles. However, multiple genotypes within buds might have been missed because we only genotyped a single anther per bud, and experimental dilutions had shown that the limits of detecting a mixtures of two alleles by our PCR method was ratio of 1:10. We therefore also genotyped all the anthers of two flower buds from each of three stems from four infected plants in which several genotypes had been detected in different stems. Again, only a single genotype was detected in each bud. Thus, if multiple genotypes are present within buds, the minority genotypes can only account for less than 10% of the teliospores in all the anthers within a bud.

**Figure 2:**
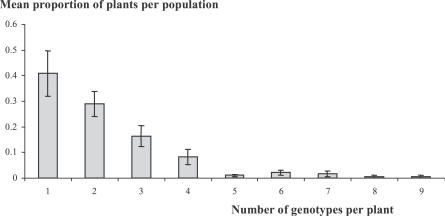
Mean Proportion of Plants per Population Having a Given Number of Fungal Genotypes Bars indicate standard errors.

### Relationship between Multiple Infections and Plant Size

We found a significant correlation between the number of genotypes and the number of stems per plant (*R* ^2^ = 0.37, *p* < 0.00001), indicating that plants with more stems are more likely to carry multiple fungal genotypes. This correlation is consistent with the findings that plants with more flowers are visited more often by pollinators [22] and that different branches from a given plant appear to be infected independently by distinct parasite genotypes in experimental field populations [19,20]. The distribution of the number of plants with a given number of genotypes deviated significantly from a Poisson distribution (*p* < 0.0001), with an excess of plants carrying many genotypes (Figure 2). Given that the number of genotypes per plant was positively correlated to the number of stems, this may reflect the distribution of the number of stems per plant, which also exhibited significantly more plants carrying many stems than would be expected from a Poisson distribution (*p* < 0.0001).

### Relatedness between Parasite Genotypes

Regression estimates based on shared alleles [23] were used to characterize the relatedness among multiple genotypes infecting a given plant. Relatedness (*r*) is an estimate of the probability of gene sharing among individuals beyond the baseline probability set by the gene's frequency in the population. Values of *r* that are significantly greater than zero are considered to represent significant relatedness, whereas negative values result when individuals are less similar than expected by chance. Such measures of relatedness are typically used in all studies on social insects within social groups where kin selection is expected to act. A few studies have shown that animal parasites were more similar within hosts than between hosts by using *Fst* [12]. Regression estimates based on shared alleles [23] have two main advantages over *Fst* for estimating relatedness among parasites: (1) *r* estimates exactly the proportion of shared alleles between individuals compared to the probability of sharing alleles by chances given the allelic frequencies in the population. It is therefore a direct measure of exactly what is important for kin selection, namely, the probability that an altruistic individual shares the altruistic allele with the individual towards whom it interacts in an altruistic manner [23]. (2) Hamilton showed that the relatedness for diploids is equal to 2*Fst*/(1 + *Fit*) [24]. *Fst* used alone can be misleading because any additional similarity to self due to inbreeding (*Fit*) would be neglected.

Parasite genotypes within plants were significantly related, both when tested over all 12 populations (*r* = 0.38 ± 0.06) and when analyzed within ten of the 12 individual populations (Figure 3). One possible explanation for such high relatedness could be isolation by distance (i.e., spatial substructure of parasite genetic diversity, which results in correlations between genetic and spatial distances). Isolation by distance has been detected within roadside populations of M. violaceum on S. latifolia [21] and was also found again in this study (not shown). We therefore tested the possibility that the significant relatedness detected here was due to isolation by distance. To this end we calculated the relatedness of genotypes present in the geographically closest pairs of diseased plants that were only infected by a single genotype. The relatedness found between strains from neighboring singly infected hosts was significantly higher than zero (*r* = 0.06 ± 0.04) but much lower than *r* in multiply infected plants, which was 0.38 ± 0.06. This indicates that isolation by distance cannot be the sole phenomenon explaining the high relatedness among strains within plants.

**Figure 3:**
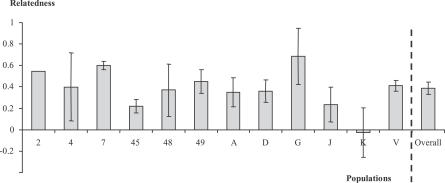
Mean Relatedness (± SE) among Genotypes within Plants, Overall, and for Each Population

A second possibility was that relatedness with multiply infected plants reflected expansion of nearly identical genotypes by selfing during parasite transmission from one branch to additional branches within the same plant, or due to crosses between multiple genotypes within plants. However, visual inspection of the microsatellite genotypes revealed that neither of these explanations were the case. Instead, most pairs of genotypes within single plants yielded three alleles for at least one locus, and the individual genotypes within a plant were most often homozygotic for different alleles at least at one locus. These observations are incompatible with selfing or crosses between resident strains, unless the crosses involved related strains. Of the 357 genotypes, only 41 could have been derived from selfing from another genotype in the same plant or from crosses between two other genotypes within the same plant. After excluding these 41 genotypes from the analysis, *r* between strains from multiply infected plants was still significant and very high (*r* = 0.35 ± 0.07). Thus, neither selfing nor crossing between resident strains seems to play a major role in the high relatedness found among parasites within plants.

We speculated that the high relatedness among genotypes within plants was due to active exclusion of genetically divergent parasite genotypes. This explanation is also strongly supported by previous experiments, which showed that sequential inoculation with more related genotypes led to a higher frequency of multiple infections than did sequential inoculation with more distant genotypes, even though similar levels of infection were obtained when strains were inoculated alone [8]. Other mechanisms, however, cannot be excluded and may contribute in addition to active exclusion, such as a higher mortality of plants carrying more divergent genotypes or a high fitness variance of the genotypes. If dissimilar genotypes had a higher average variance in fitness, one genotype might indeed predominate more rapidly than when the genotypes are more similar. These hypotheses will be tested in future work following experimental populations.

### Relationship between Multiple Infections and Diversity of Parasite Populations

No significant correlation was found between the mean number of genotypes per plant and the size of the population. Instead, and somewhat surprisingly, we found a significant negative correlation over all 12 plant populations for the mean number of genotypes per plant versus the genetic diversity of the parasite population (Figure 4; *r* = −0.62, *p* < 0.03). This observation was surprising because a naive assumption might have been that multiple infections would be more frequent in populations with higher parasite genetic diversity. We argue instead that multiple infections in more diverse parasite populations occur less frequently due to active exclusion. In populations with higher numbers of parasite genotypes, the frequency of multiple infections is reduced due to a higher probability that a strain deposited on an infected plant is sufficiently genetically different from the resident genotype to be excluded. The negative correlation between the mean number of genotypes per plant and the genetic diversity of populations thus provides further support for relatedness-dependent competitive exclusions by M. violaceum.

**Figure 4:**
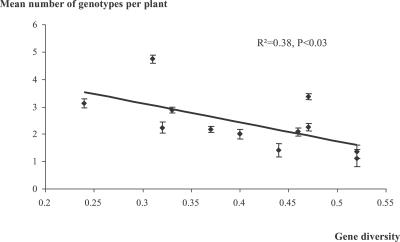
Mean Number of Parasite Genotypes per Plant (± SE) Plotted against the Genetic Diversity within Populations

### Putative Mechanisms of Exclusion between Fungal Strains

Active genotype exclusion might result from the production of specific toxins against competitors [25] or from mechanisms of vegetative incompatibility, such as the death of hyphal cells after fusion of two genetically different genotypes [26]. Unfortunately, these mechanisms are difficult to test experimentally in *Microbotryum*, because hyphae cannot be cultured in vitro and hyphal interactions are not readily observable in host tissues. However, it is very likely that different strains within a plant come in close contact and can compete, even if we only detected them in different stems. When single strains are inoculated onto a plant, disease is only evident during the first year on the branch receiving the inoculation, and sometimes the branches next to the inoculated flower. However, all the flowers are usually diseased once new branches and flowers grow the next year. This is thought to reflect invasion of all the meristematic tissues of the plant by the fungus during the winter [27]. When a plant is infected by multiple strains, different genotypes are thus likely to compete directly for colonization of meristems during the following winter.

### Expected Consequences on Virulence

Virulence is expected to be higher in systems where multiple infections are frequent and faster rates of host exploitation lead to a transmission advantage [2]. In sterilizing parasites such as M. violaceum, whose major impact on host fitness is the prevention of reproduction, castration is also expected to be maximal [28]. Therefore, it does not appear surprising that M. violaceum exhibits very high virulence, and plants are usually completely sterile one year after the first infection. The frequent occurrence of multiple infections in this system may have been one of the factors selecting for high virulence. In contrast, relatedness-dependent competitive exclusion in M. violaceum appears to restrict multiple infections to more closely related genotypes and should thus lessen the severity of evolutionary conflicts between competing parasite genotypes. Such contrasting selective forces have resulted in the variable virulence dependent on within-host dynamics in other systems [29]. The host plants for M. violaceum can recover with substantial probabilities [30,31], which would be expected to result in variable virulence if the probability of dying before recovering varied between plants with single or multiple infections. For example, if the strains in multiple infections were to produce more spores, they might drain host resources more rapidly and make the plant die faster. Variability in teliospore production among strains has been reported [32]. The presence of multiple genotypes might also directly affect the probability of plant recovery. We will follow individual plants in experimental populations in future experiments to investigate whether the probability of dying and/or recovering depends on the number of genotypes within a host plant and/or their relatedness.

### Conclusion

This study shows that there is a high and significant relatedness among fungal parasite genotypes in multiply infected host plants. Together with a previous experimental study [8], our results strongly suggest that the significant relatedness of parasites within hosts is due to an active mechanism of exclusion. Microgeographic structure, selfing, and crosses on the host probably also contribute to the high relatedness of parasite genotypes within host plants, but to a lesser extent. Theoretical models and experimental studies have shown that competition between parasites in multiple infections should be weaker when parasites within a given host are more closely related [3–7]. Our results show that the biased occurrence of related parasite genotypes within hosts can indeed be found in natural populations, thus providing empirical support for previous theoretical considerations.

## Materials and Methods

### Teliospore collection and populations.

Teliospores of M. violaceum were collected in the fall of 2004 from 12 roadside populations of S. latifolia in the Essonne region (France). These were also included in the populations sampled by Giraud [21], who analyzed one single flower bud per plant. Here, we collected one bud from each of the stems of every diseased plant in the populations. Sampled populations contained a mean ± standard error (SE) of 18.3 ± 3.0 diseased plants, having a mean ± SE of 5.7 ± 0.3 stems. As far as we could assess, the populations grew in very similar environments (roadsides), with similar levels of disturbance.

### Genotyping and detection of multiple genotypes.

To estimate the number of different genotypes per plant, we collected and genotyped all the infected individuals in each studied population. A single bud was genotyped per branch. To test for the presence of more than one genotype within single floral buds, we separately genotyped all the ten anthers for each of two buds belonging to each of three stems. This test was performed on a total of four plants that had been previously scored as having multiple fungal genotypes in different stems. Samples were stored and DNA extracted as in Giraud [21]. We scored six microsatellite loci whose polymorphism is sufficiently high in the studied populations for distinguishing genotypes: L14, L17, L18 [33], SL8, SL12, and SL19 [34], using the same protocol as Giraud [21]. All these markers are nuclear and can yield diploid genotypes in *Microbotryum* teliospores. For ten plants with identical microsatellite genotypes, we confirmed that their genotypes were identical by AFLP that was performed as in López-Villavicencio et al. [17].

### Population genetic analyses using microsatellite data.

When multiple samples of an identical genotype were found within a plant, a single sample was kept for the subsequent genetic analyses. This provides conservative estimates of relatedness because multiple identical genotypes could potentially represent the same fungal individual present in multiple stems. The unbiased genetic diversity of Nei [35] was computed using FSTAT [36].

Relatedness estimates were computed in a given population based on the allelic frequencies estimated within that population. Standard errors of *r* were obtained by jack-knifing over loci. Relatedness calculations were performed using RELATEDNESS 5.0 (http://www.gsoftnet.us/GSoft.html).

## Abbreviations

AFLP: amplified fragment length polymorphism
SE: standard error
